# Peer Effects on Real-Time Search Behavior in Experimental Stock Markets

**DOI:** 10.3389/fpsyg.2021.635014

**Published:** 2021-07-12

**Authors:** Xuejun Jin, Xue Zhou, Xiaolan Yang, Yiyang Lin

**Affiliations:** ^1^School of Economics, Zhejiang University, Hangzhou, China; ^2^School of Business and Management, Shanghai International Studies University, Shanghai, China

**Keywords:** selling stocks, peer effects, feedback, risk attitude, search behaviors

## Abstract

It is a well-documented phenomenon that individuals stop searching earlier than predicted by the optimal, risk-neutral stopping rule, leading to inefficient searches. Individuals' search behaviors during making investment decisions in financial markets can be easily affected by their peers. In this study, we designed a search game in a simplified experimental stock market in which subjects were required to search for the best sell prices for their stocks. By randomly assigning subjects into pairs and presenting them with real-time information on their peers' searches, we investigated the effects of peers' decisions on search behaviors. The results showed that two subjects in the same group with real-time peer information learned and engaged in similar search behaviors. However, this peer effect did not exist when subjects had access to feedback information on the ex-post best response. In addition, we found that the presence of information about peers' decisions alone had no significant impact on search efficiency, whereas access to both information on peers' decisions and feedback information significantly improved subjects' search efficiency.

## Introduction

Search behavior has received much attention in various areas of economics, such as job searches in labor economics (Lippman and Mccall, 1976; Braunstein and Schotter, 1982; Cox and Oaxaca, 1989), shopping choices in consumer economics (Stigler, 1961; Kogut, 1990), investment decisions in financial economics (Sirri and Tufano, 1998) and international trade in international economics (Besedes, 2008). In the process of selling stocks in the financial market, individuals often need to search for information about the stock value and are typically confronted with situations where they must either accept an attractive offer or defer the decision in hopes of receiving a better price. In general, longer searches lead to more valuable information but increase search costs. The optimization of the search problem can be challenging for unsophisticated or boundedly rational individuals due to a lack of experience and limited cognitive abilities.

While making complicated investment decisions, the search behaviors of investors may be influenced by the choices of their peers due to limited cognitive abilities, lack of experience, and the high cost of thinking (Lieber and Skimmyhorn, 2018). The current literature on the links between peer effects and investment decisions has focused on purchasing risky assets rather than on selling stocks, although selling decisions are an important part of an investment strategy and determine the final income of investors. Investors simultaneously engaging in large-scale selling behaviors can cause substantial stock price crashes and substantial consequent damage to the financial market. Media coverage and academic studies attribute this phenomenon to herd behavior (Bikhchandani and Sharma, 2001; Hirshleifer and Teoh, 2003; Jegadeesh and Kim, 2007). Panicked investors are easily affected by one another and tend to imitate the selling behavior of each other and stop searching for their reservation prices, thus accelerating a drop in stock price. Therefore, it is particularly important to investigate peer effects in the search process of selling stocks.

However, identifying the effect of the decision of one's peers on one's own through empirical analysis is notoriously difficult because information on peers' decisions in real-world financial markets contains a mix of various kinds of information, and it is hard to tell whether investors are affected by or even notice others' decisions. Experimental methods of constructing an artificial stock market can help overcome this challenge and control the effects of confounding information.

Although the optimization problem underlying the search task is rather difficult to solve, the simple structure of the search task still makes it attractive for studying many substantive issues experimentally and has proven to be useful in behavioral research in psychology and economics (Schunk and Winter, 2009). Numerous experimental studies have performed the search task in many contexts, such as a job seeker searching for a wage offer (Brown et al., 2011) or a seller searching for a selling price (Einav, 2005). In this study, we focus on search behavior in selling stocks and deliberately neglect factors other than peer effects that may also affect this behavior, such as the market price of the asset. We want to ensure that the task is readily understood by subjects to focus on our main interest, peer effects, and isolate other possible effects resulting from the complexity of the task.

This paper aimed to design a laboratory experiment to test for peer effects in search behaviors related to selling stocks. Furthermore, we examine peer effects again in the presence of feedback information related to a previous trial. We seek to determine whether peer effects are mitigated when individuals are provided with useful information. If so, we may infer that peer effects work primarily through the social learning channel and that individuals learn from their peers at least partially because they lack information about the financial market. Finally, it is particularly important to identify the effect of observing peers' decisions on search efficiency. As previously stated, subjects are likely to spend too little time searching and violate the optimal stopping rule. We investigate whether access to information about peers' decisions helps reduce subjects' inefficient search behaviors in the financial market.

In this experimental study, we designed a simplified stock market. Subjects in both pair and single treatments were required to complete a sequential search task that consisted of searching for the proper price to sell their stocks with/without information on their peers' decisions. Comparing the search behaviors of true pairs in the pair treatment with those of simulated pairs drawn from the single treatment, we tested the existence of peer effects in stock price search behaviors in a controlled laboratory setting, which allowed us to eliminate confounding factors that may interfere with subjects' decisions. Additionally, we conducted a pair-with-feedback treatment to investigate the effect of feedback information on peer effects. We would like to know whether subjects still learn from their peers if they have access to valuable information.

This study makes three contributions to the literature on peer effects in investment decisions. First, most studies of peer effects have focused on how peers' decisions affect individuals' decisions to buy risky assets (Bursztyn et al., 2014; Delfino et al., 2016) and do not consider their effects on selling assets. We fill this gap by testing for peer effects in search behaviors related to selling stocks. We found that peer effects were significant in search behaviors related to selling stocks under the pairwise condition, indicating that members in the same group learned from each other and adopted similar behaviors during the selling process. Second, individuals may refer to the actions of peers due to a lack of information about the financial market. Therefore, we provided subjects with feedback information about the ex-post best response in the pair-with-feedback treatment to determine whether access to valuable information mitigates peer effects. The results support our hypothesis and show that feedback information about the ex-post best response moderates the effects of peers in search behaviors. Subjects ignored their peers' decisions when they had access to feedback information about the ex-post best response. Third, we identified the effect of various information conditions in the three treatments on accepted price and search efficiency. Controlling for risk attitudes, which is the key determinant of search behavior found by the existing literature, the regression results for accepted prices and optimal search behaviors confirm only the substantially positive effect of access to the decisions and feedback information on search efficiency of both peers. As subjects did in the pair treatment, learning only from the decisions of peers did not improve the efficiency of the search.

The remainder of the paper is organized as follows. Section 2 briefly reviews the related literature. Section Methods describes the experimental design, procedures, and methods of data analysis. Section Results presents the results of our experiment, and section Discussion and Conclusion discusses the results.

## Literature Review

To our knowledge, this study is the first to provide evidence on peer effects in investment decisions related to selling stocks. Our study mainly relates to two aspects of the literature: search behaviors and peer effects in investment decisions.

### Search Behaviors

Search behaviors play an important part in many situations, including making investment decisions. Investors must consider a massive amount of information and search for the best price for buying or selling assets. Evidence from Sirri and Tufano (1998) supports the material impact of search costs on individuals' mutual fund purchase choices.

Extensive experimental research has examined search behaviors under various contexts and observed the common phenomenon of early stopping of search behavior compared to the stopping in an optimal, risk-neural search strategy. Subjects tend to stop searching earlier than is optimal and accept lower prices than the optimal reservation point. Ambiguity over the price distribution (Asano et al., 2015) and over the waiting time for offers to arrive (Brown et al., 2011) can lower individuals' confidence about the future and decrease their reservation prices, discouraging them from searching for longer durations. Risk attitude and cognitive ability are two major determinants of search behaviors. First, abundant evidence has shown that search rules derived under the assumption of risk aversion better describe search behaviors than those derived under the risk-neutral assumption (Cox and Oaxaca, 1989; Sonnemans, 1998). Risk-averse individuals tend to stop searching too early to avoid making decisions at risk. The higher the level of risk aversion, the less likely an individual is to continue a risky search. Second, heuristics adopted due to limited cognitive abilities also impact search behavior and may lead to larger deviations from optimal search strategies. The existing literature has suggested that limited cognitive ability is the direct reason individuals cannot compute optimal reservation prices and instead follow simple heuristics (Moon and Martin, 1990; Schunk and Winter, 2009).

### Peer Effects in Investment Decisions

Peer effects are frequently observed in various financial decisions, including investment decisions (Bursztyn et al., 2014; Delfino et al., 2016), retirement savings (Beshears et al., 2015), and charitable giving (Lieber and Skimmyhorn, 2018). The existing literature has identified two principal channels underlying peer effects (Cooper and Rege, 2011; Bursztyn et al., 2014; Lahno and Serragarcia, 2015): social learning and social utility. According to the social learning channel, one may learn from information inferred from peers' choices, highlighting the significance of social learning in behavior modification. According to the social utility channel that builds upon social comparison theory (Festinger, 1954), one's utility may be directly enhanced by peers searching for the same length of time or accepting the same price.

Previous research on peer effects in investment decisions, which relates most directly to our study, has focused on purchasing risky assets (Bursztyn et al., 2014; Delfino et al., 2016). Bursztyn et al. (2014) and Delfino et al. (2016) both reported significant peer effects in decision-making related to risky investments. Bursztyn et al. (2014) explored the mechanisms underlying peer effects and confirmed the effects of social learning and social utility channels. Delfino et al. (2016) investigated the effect of the three main features of a risky investment task on peer effects in investment choices, including time pressure, social information, and uncertainty. They found that peer effects are greater for investments with higher time pressure and social information, representing average group behavior and risk more than uncertainty. Based on this literature, it is reasonable to hypothesize that peer effects exist in the search process of buying risky assets and the search process of selling risky assets.

## Methods

### Theory and Background

The theoretical foundation for the experimental design is a standard finite-horizon sequential search problem in which an agent pays a constant cost for each new observation (Einav, 2005; Holt, 2005; Brown et al., 2011; Asano et al., 2015). Each observation is independently drawn from a probability distribution. The agent has complete information about the observation distribution and the fixed search cost. In each trial of the search problem, the agent searches for the price to sell a good and decides when to stop searching. If the agent accepts an arriving observation, one search trial is concluded, and the accepted observation is accepted for selling the good. If the agent rejects an arriving observation, he/she pays a constant cost for another observation. The agent continues to search until he/she accepts an observation. Recall is not allowed.

In this well-known search problem, a perfectly rational, risk-neutral agent employs the strategy of setting a reservation price, the price at which the expected benefit of another search round is equal to the search cost (Kohn and Shavell, 1974; Holt, 2005), i.e., the reservation price *R* is the solution to Equation (1).

(1) $$\begin{array}{r} c=\left[1-F\left(R\right)\right]E_{F}\left(p-R|p\geq R\right) \end{array}$$

where *c* is the constant search cost, *p* is the sampled price and *F*(·) is the cumulative distribution function of prices. When we set *c* = 20 and *p*~*U*(0, 1, 000) in the experiment, we obtain *R* = 800. Suppose the current draw is 800; there is a 4/5 chance that the next draw is 800 or below, in which case the net gain is 0, and the expected value of the gain is (4/5) ^*^ 0 = 0; there is a 1/5 chance that the next draw is more than 800, in which case the net gain on average is half of the distance from 800 to 1,000, i.e., 100, and the expected value of the gain is (1/5) ^*^ (100) = 20. Therefore, the total expected benefit of another search is 20, equal to the search cost *c*. For any risk-neutral subject, the optimal search strategy is to continue to search until a draw of more than 800 appears.

### Experimental Design

**Figure 1:**
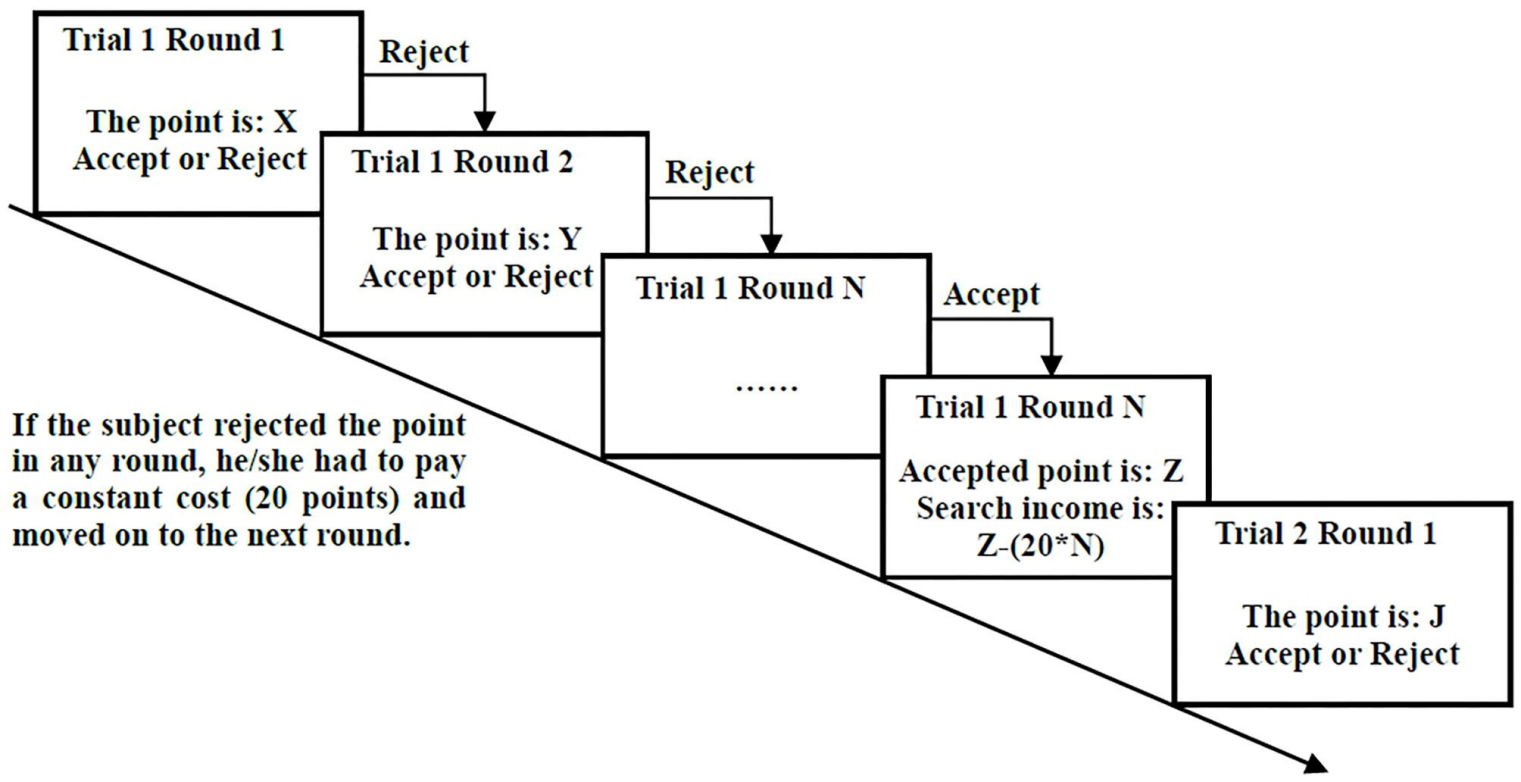
Search task.

The experimental design implemented the above search problem with *c* = 20 and *p*~*U*(0, 1, 000). We repeated the search task for 40 trials with different price realizations to obtain sufficient observations for a reliable analysis and to control the length of the experiment to avoid exhausting our subjects. In each trial, the subjects searched for the selling price for one stock portfolio from a uniform distribution (0, 1,000). In the first round of each trial, the price of the stock portfolio was drawn randomly by a computer from a uniform distribution with a lower bound of 0 and an upper bound of 1,000.^1^ After observing the given point^2^ on the screen, the subject was expected to click either the “accept” or the “reject” button. If the subject accepted the price point, the current trial was concluded, and the accepted price for selling one share of the stock portfolio was converted into a payment. If the subject selected the reject button, he/she had to pay a constant search cost of 20 points and then moved on to the next round, in which another point was drawn from the same uniform distribution. The subject continued to search in this manner until he/she accepted a point. The process of the search task is shown in Figure 1.

At the end of the experiment, a random trial was selected for payment, and 100 experimental points could be converted to 1.50 CNY (~0.21 USD^3^) in this game. For example, in one trial, a subject rejected the points offered in the first three rounds and then accepted the point of 852 in the fourth round. As the search duration was four rounds, the total search cost was 80 points, and the search payoff was 11.58 CNY (~1.64 USD^3^).

After the search task, the subjects also participated in an investment game and completed a questionnaire to gather data on risk attitudes and personal information, respectively. The investment game adopted a standard design for measuring risk attitudes based on Charness and Gneezy (2010). Subjects were required to choose an amount 0 ≤ *X* ≤ 100 to allocate between a risky asset that had an equal probability of paying either 0 or 2.5*X* and a risk-free saving 100−*X*≥0 that paid out with certainty. Allocating a higher percentage of the investment to risky assets generally indicates a stronger preference for risk-taking.

Our experiment consisted of three treatments that differed only in the search task, as described in Table 1. We varied the peer conditions in the single and pair treatments to examine our main interest, peer effects. We also provided paired subjects with additional feedback information about the ex-post best response in the pair-with-feedback treatment to investigate the effect of feedback information on peer effects in stock price search behaviors.

**Table 1:** Experimental design and number of subjects.

**Treatment**	**Single/paired**	**Feedback**	**Male subjects**	**Female subjects**	**Mean age**
Single	Single	No	26	23	21.84
Pair	Paired	No	9	11	22.30
Pair-with-feedback	Paired	Yes	7	9	23.81

In all three treatments, the subjects were asked to complete a sequential search task. The difference between the single and pair/pair-with-feedback treatments lay in whether the subject was alone or randomly grouped with another participant and could see his/her partner's real-time search information on the computer screen. The peer's information included which round the peer was currently in and what his/her decision was in that round. Subjects in the pair-with-feedback treatment not only saw the real-time information of their group members but were also notified whether they had stopped the search earlier or later than the optimal search duration at the end of each trial. This feedback information about the best response in the last trial, which was shown on the computer screen, was presented as follows:

“*Compared with the optimal search duration, you stopped searching too early/at the same time/too late.”*

The optimal search duration was the number of search rounds derived from the optimal, risk-neutral stopping rule and the predetermined random draws from the point distribution. Based on the theoretical background, the optimal search strategy is to continue to search until a draw of more than 800 points appears, and this stopping rule determines the optimal search duration.

Table 1 reports detailed information about the sexes and mean ages of the subjects by treatment group.

### Participants and Procedure

The 85 participants were recruited from Zhejiang University. They were randomly assigned to one of the three treatment groups: single (*n* = 49, 23 females), pair (*n* = 20, 11 females), and pair-with-feedback (*n* = 16, 9 females).

The experiment was conducted with z-Tree software (Fischbacher, 2007). At the beginning of the experiment, each subject was provided with written instructions. The subjects were informed that (i) they would not incur any losses from the search task; (ii) they would earn a show-up fee of 20.00 CNY (~2.83 USD^3^); and (iii) other payoffs were determined by the decisions they made in the experiment. After a public reading of the instructions, three pilot trials were conducted to allow subjects to practice the search task. Then, the experiment proceeded with a 40-trial search task, an investment game, and a questionnaire containing 14 questions about personal information such as sex, age, major, place of origin, household income, and consumption expenditure.

The experiment lasted ~35 min, and the average payoff was 39.38 CNY (~5.57 USD^3^). Each participant was required to provide written informed consent, as approved by the Zhejiang University ethics committee.

### Data Analysis

We tested whether the search behaviors of the subjects during the process of selling stocks were influenced by their peers and whether feedback information about the optimal search duration moderated the peer effects.

First, following Falk and Ichino (2006), we adopted the between-minus-within standard deviations (for simplicity, denoted by “*Stdp*” hereafter) of subjects' average search durations, average accepted prices, and average incomes^4^ to measure the peer effects. In this analysis, between-minus-within variance (denoted by “*Varp*”) was equal to between-pairs variance (denoted by “*Varb*”) minus within-pairs variance (denoted by “*Varw*”), as shown in equation (2). *X* refers to the three characteristics of search behaviors (search durations, denoted by *D*; accepted prices, denoted by *P*; and incomes, denoted by *I*). *Stdp* was then calculated as the square root of *Varp* in equation (3):

(2) $$\begin{array}{rcl} Varp_{X} & = & Varb_{X}-Varw_{X} \end{array}$$

(3) $$\begin{array}{rcl} \text{and }Stdp_{X} & = & \sqrt{Varp_{X}}=\sqrt{Varb_{X}-Varw_{X}} \end{array}$$

*Varb* was defined as the variance of the average search variables *X*_*j*_ of group *j*, where the total number of groups was denoted by *m*:

(4) $$\begin{array}{r} Varb_{X}=\sqrt{\frac{\overset{m}{\underset{j=1}{\sum }}{\left(X_{j}-{\bar{X}}_{j}\right)}^{2}}{m}} \end{array}$$

*Varw* was then calculated as the difference between the total variance at the individual level (denoted by “*Var*”) and *Varb*:

(5) $$\begin{array}{rcl} Var_{X} & = & \sqrt{\frac{\overset{m}{\underset{j=1}{\sum }}\overset{2}{\underset{i=1}{\sum }}{\left(X_{ji}-{\bar{X}}_{ji}\right)}^{2}}{2m}} \end{array}$$

(6) $$\begin{array}{rcl} \text{and }Varw_{X} & = & Var_{X}-Varb_{X} \end{array}$$

The basic logic was that in the absence of peer effects, which means that one member's decision does not affect the search behavior of the subjects, the variance in the search variables within pairs (*Varw*) should be identical to the variance generated by any simulated configuration of pairs constructed from the subjects in the single treatment. If the peer effects were positive, more similar search behaviors should generate smaller *Varw* values and higher *Stdp* values. Accordingly, we separately examined the peer effects in the pair and pair-with-feedback treatments with simulated configurations of pairs constructed from subjects in the single treatment and drew the opposite results, as discussed in more detail in section Results.

Statistical analyses, including the calculations of standard deviations, simulations, and kernel density plots, were performed using R 3.5.1.

## Results

Table 2 summarizes the search behaviors across the three treatments, including average search duration, average accepted price, and average income. On average, the subjects in the pair-with-feedback treatment searched for a longer time, accepted a higher price (higher than the optimal reservation point, 800), and earned a higher income than the subjects in the single and pair treatments. Regarding peer effects under different information conditions, the *Stdp* of the pair treatment was higher than that of the pair-with-feedback treatment, suggesting that members of pairs in the pair treatment had more similar search behaviors than members of pairs in the pair-with-feedback treatment.

**Table 2:** Mean and *Stdp* of search variables in each treatment.

		**Average search duration**	**Average accepted price**	**Average income**
Single	Mean	5.801	786.558	10.058
		(3.333)	(157.205)	(2.158)
	*Stdp*^a^	(−0.996, 0.917)	(−65.751, 65.079)	(−46.444, 47.706)
Pair	Mean	5.478	771.869	9.935
		(3.103)	(156.296)	(2.127)
	*Stdp*	0.740	50.970	34.830
Pair-with-feedback	Mean	7.070	854.273	10.693
		(3.472)	(109.031)	(1.811)
	*Stdp*	−0.064	−5.665	−2.683

*Standard deviations in parentheses*.

a *We generated simulated pairs from the single treatment twice. The Stdp here are the between-minus-within standard deviations of 654,729 simulated configurations, as discussed in section Peer Effects in Search Behavior. Using the Stdp of 202,702 simulated configurations in section Effect of Feedback Information on Peer Effects did not change the overall results*.

Following Falk and Ichino (2006), we generated 654,729,075 possible configurations of 10 pairs of 20 randomly chosen subjects from the single treatment and computed the *Stdp* for 654,729 randomly chosen configurations.^5^ The minimum and maximum values of *Stdp* for these selected simulated configurations are reported in Table 2.

### Peer Effects in Search Behavior

Our main concern was peer effects in search behaviors related to selling stocks, as revealed by the similar search behaviors of pairs in the pair treatment group. In the absence of peer effects, the *Stdp* of the pair treatment should be identical to that generated by any simulated group of pairs from the single treatment. In the presence of peer effects, the more similar search behaviors of true pairs in the pair treatment should generate significantly higher *Stdp*.

**Figure 2:**
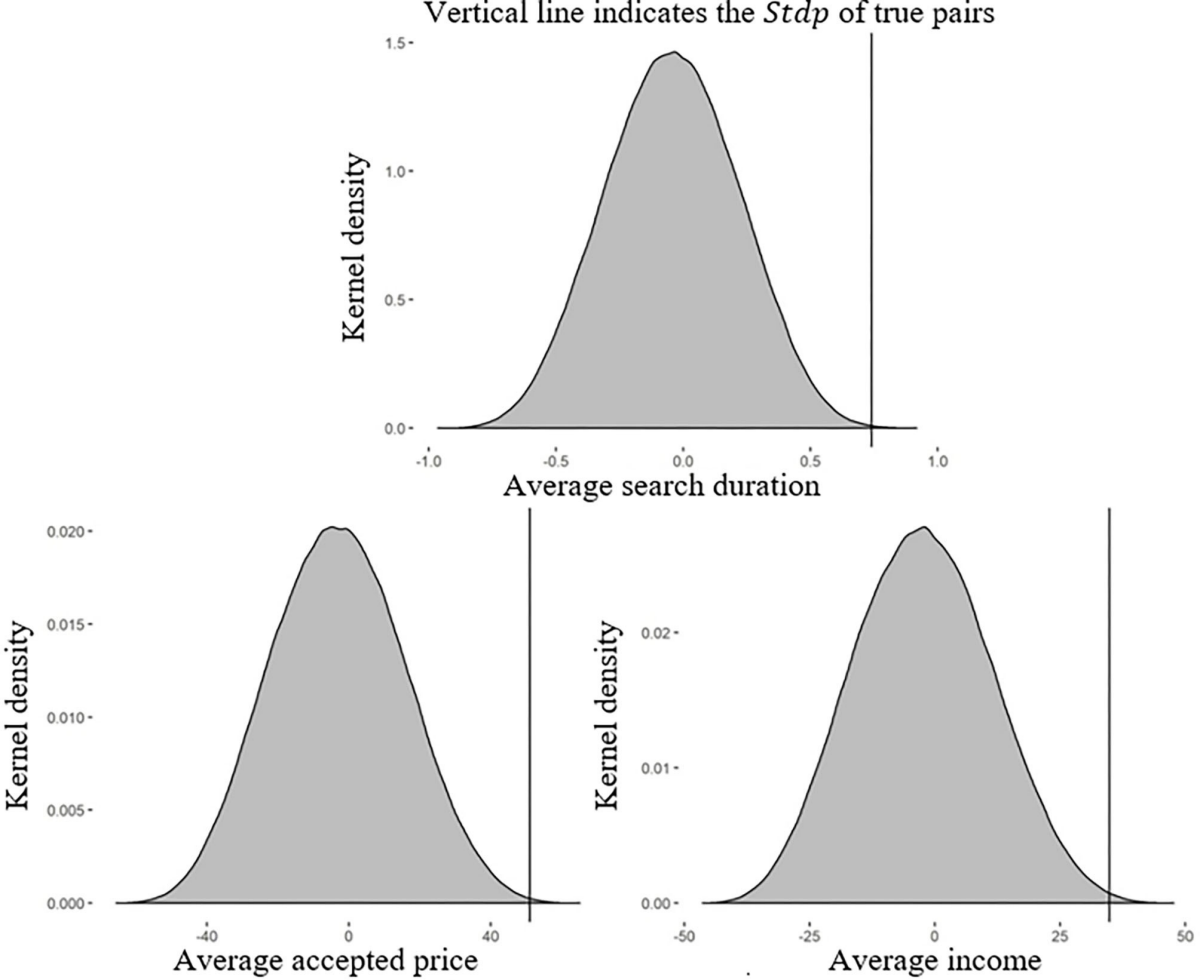
Kernel density of the *Stdp* of the true pairs in the pair treatment and the simulated pairs in the single treatment. In the absence of peer effects, the differences between the *Stdp*s of the pair and single treatments should not be systematically positive or negative. By contrast, *Stdp* values for the pair treatment (the vertical lines) higher than 99.5% of those generated by the simulated pairs in the single treatment (the distributions) in our experiment indicate significant peer effects in the pair treatment.

Figure 2 plots the kernel density of the *Stdp* of the true pairs from the pair treatment and 654,729 simulated configurations drawn from the single treatment. The curved lines above the gray area represent the kernel density of the *Stdp* of the simulated configurations, and the vertical lines indicate the *Stdp* of the true pairs. The *Stdp*_*D*_ of the simulated configurations ranged from −0.996 to 0.917 (*Stdp*_*P*_, −65.751 to 65.079; *Stdp*_*I*_, −46.444 to 47.706), whereas the *Stdp*_*D*_ of the true pairs, represented by the vertical line, was 0.740 (*Stdp*_*P*_, 50.970; *Stdp*_*I*_, 34.830).

As shown in Figure 2, the *Stdp*_*D*_ of the true pairs was higher than 99.95% of the *Stdp*_*D*_ values derived from simulated pairs drawn from the single treatment. If there was no peer effect, the *Stdp*_*D*_ of the true pairs should have been close to the median of the simulated pairs. The likelihood of such a large difference in the standard deviation being generated in the absence of peer effects was extremely low (i.e., <0.05%), suggesting that we could reject the null hypothesis with a high level of confidence.

Similarly, the *Stdp*_*P*_ of the true pairs was higher than 99.91% of the *Stdp*_*P*_ values of the simulated pairs. The *Stdp*_*I*_ of the true pairs was higher than 99.75% of the *Stdp*_*P*_ values of the simulated pairs. As higher *Stdp* values indicate more similar search behaviors, we concluded that members in the same group in the pair treatment had significantly similar search behaviors regarding search duration, accepted price, and income, demonstrating the existence of peer effects.

**Result 1:** Members of true pairs in the pair treatment had significantly more similar search behaviors than members of simulated pairs drawn from the single treatment. This demonstrates that peer effects are significant in the stock price search process.

### Effect of Feedback Information on Peer Effects

We seek to determine whether, in the presence of feedback information about optimal search duration, individuals continue learning from their peers or ignore information on their peers in favor of the feedback information. The latter case would highlight the social learning mechanism through which peer effects work. Therefore, we tested the existence of peer effects in the pair-with-feedback treatment as follows.

**Figure 3:**
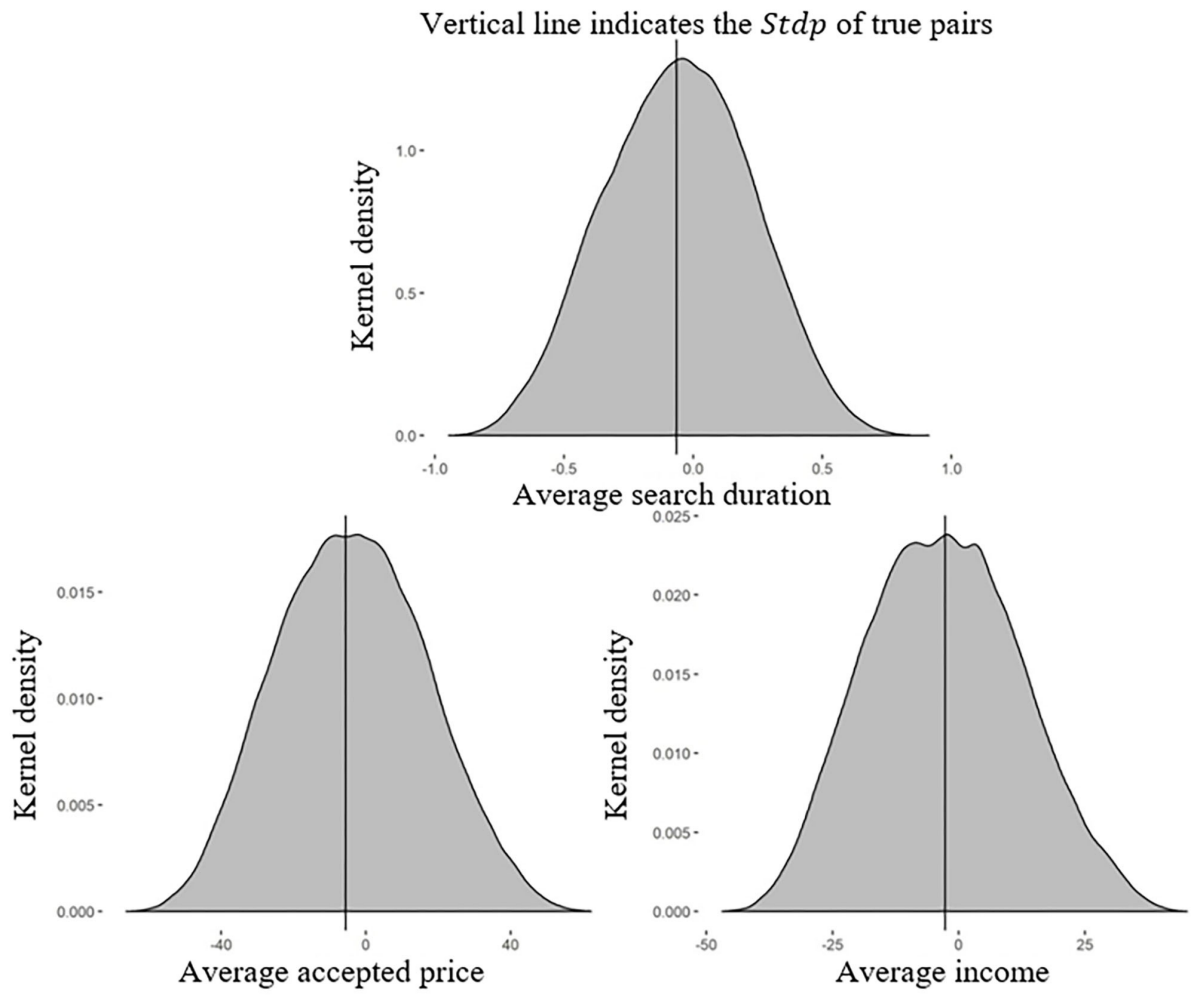
Kernel density of the *Stdp* of the true pairs in the pair-with-feedback treatment and the simulated pairs in the single treatment. The differences between the *Stdp*s of the pair-with-feedback and single treatments were approximately symmetric around zero, which was exactly what we would expect in the absence of peer effects.

As before, we generated 2,027,025 possible configurations of 8 pairs made up of 16 randomly chosen subjects from the single treatment and computed the *Stdp* for 202,702 randomly chosen configurations. Figure 3 plots the kernel density of the *Stdp* of the true pairs in the pair-with-feedback treatment and the 202,702 simulated configurations drawn from the single treatment. The *Stdp*_*D*_ of these simulated configurations ranged from −0.948 to 0.913 (*Stdp*_*P*_, −66.402 to 62.239; *Stdp*_*I*_, −46.947 to 45.223). The vertical line identifies the *Stdp*_*D*_ of the true pairs as −0.064 (*Stdp*_*P*_, −5.665; *Stdp*_*I*_, −2.683).

Figure 3 shows that the *Stdp*_*D*_ of the true pairs was higher than 48.24% of the *Stdp*_*D*_ values of the simulated pairs in the single treatment (47.37% for *Stdp*_*P*_ and 51.13% for *Stdp*_*I*_). The differences, approximately zero, were not significantly positive or negative. This was the expected result in the absence of peer effects. Therefore, we could not reject the null hypothesis. The results suggested that individuals ignored their peers' decisions when observing feedback information about the ex-post best response.

**Result 2:** Feedback information about the optimal search duration eliminated the peer effects in stock price search behaviors. Individuals stopped learning from their peers when they have access to this feedback information.

### The Efficiency of Search

**Figure 4:**
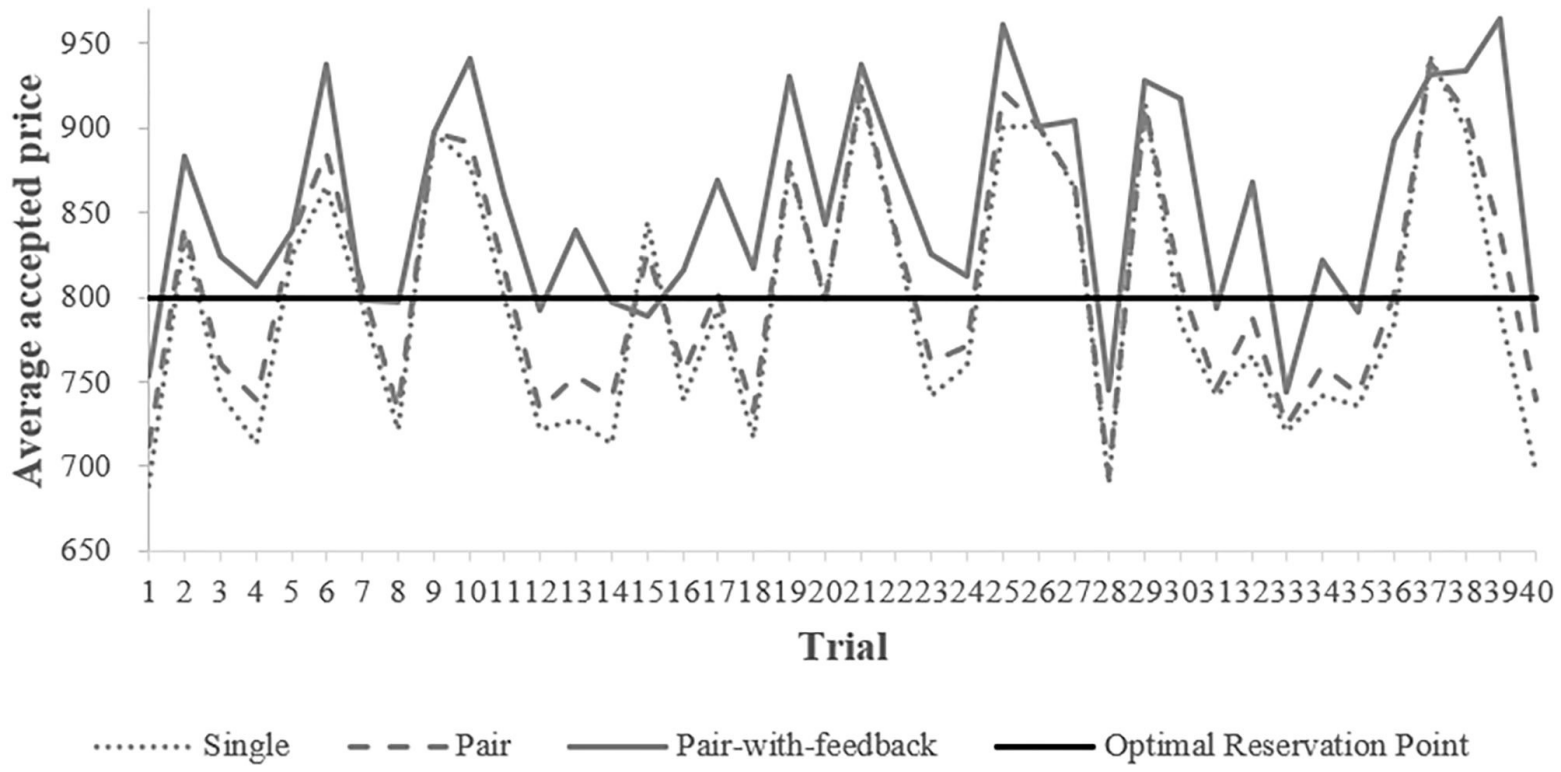
Time series of average accepted prices.

As indicated in Table 2, on average, only the accepted price of the pair-with-feedback treatment was higher than the optimal reservation point, 800. The means of the accepted prices of the single and pair treatments did not show much difference at first glance. Figure 4 depicts the evolution of the average accepted prices across treatments over the 40 trials of the search task. The average price accepted by subjects from the pair-with-feedback treatment (the gray, solid line) substantially exceeded the optimal reservation point (Wilcoxon signed-rank tests, *p* < 0.001) and the average prices accepted in the other two treatments (Wilcoxon rank-sum tests, *p* = 0.0004 for the single treatment and 0.004 for the pair treatment) in most of the trials. However, in the single and pair treatments, the average accepted prices were below the optimal reservation point in nearly half of the trials. We found no significant difference in the average accepted prices between the single and pair treatments (Wilcoxon rank-sum tests, *p* = 0.294). These results indicate that the subjects searched for higher selling prices only when observing both feedback information and their peers' decisions.

Higher accepted prices within the pair-with-feedback treatment could have two explanations. First, the subjects increased their reservation prices by learning from the feedback information, their peers' decisions, or both. Second, the subjects from the pair-with-feedback treatment had higher reservation prices because they inherently had higher preferences for taking risks, which has been identified as a determinant of search behavior by numerous studies (Cox and Oaxaca, 1989; Sonnemans, 1998; Yang et al., 2018).

As shown in Figure 4, the differences appeared from the beginning of the experiment between the pair-with-feedback treatment and the single/pair treatment, so we cannot rule out the second possibility of differences in risk attitudes. To increase the robustness of our conclusions, we employed ordinary least squares to estimate the effect of the informational conditions in the three treatments on accepted prices, controlling for the effects of individual characteristics, including risk preferences, age, and sex.

The regression results are presented in the first two columns of Table 3. We regressed each subject's accepted price in each trial on two treatment dummies. The dependent variable was *Accepted price*, which was the value of the price that the subject accepted in the current trial of the search task. Treatment effects were estimated using two dummy variables. The dummy variable *pair treatment* was equal to 1 for the subjects in the pair treatment in which real-time peer decisions could be observed, and the dummy variable *pair-with-feedback treatment* was equal to 1 for the subjects in the pair-with-feedback treatment in which both real-time peer decisions and feedback information about the optimal search duration were available. These two dummy variables capture the treatment effects relative to the single treatment in which the subjects received no information about their peers' decisions or feedback. The control variables included age and sex for individual-specific characteristics and trial dummies for time trends.

**Table 3:** Regression results for search efficiency.

**Dependent variable**	**(1) Accepted price**	**(2) Accepted price**	**(3) Optimal search**	**(4) Optimal search**
Pair treatment	−12.337	−14.283	−0.087	−0.099
	(24.743)	(24.176)	(0.147)	(0.147)
Pair-with-feedback treatment	75.393^***^	73.512^***^	0.534^***^	0.523^***^
	(17.290)	(17.221)	(0.124)	(0.121)
Risk preference		71.858^*^		0.459^*^
		(38.004)		(0.238)
Age	−3.443	−4.518	−0.037	−0.044^*^
	(4.000)	(3.954)	(0.024)	(0.024)
Sex (=1 if female)	−9.404	4.049	−0.024	0.062
	(18.603)	(20.321)	(0.116)	(0.121)
Trial	1.259^***^	1.259^***^	−0.0003	−0.0003
	(0.287)	(0.287)	(0.002)	(0.002)
Constant	840.337^***^	815.212^***^	0.688	0.528
	(90.049)	(91.698)	(0.543)	(0.560)
*N*	3,400	3,400	3,400	3,400
(Pseudo)^a^ R^2^	0.048	0.065	0.008	0.011
*P*-value	0.0000	0.0000	0.0001	0.0000

*Robust standard errors clustered by subject are in parentheses*.

*** *p < 0.01;*

* *p < 0.10.*

*The regressions presented in columns (1) and (2) were estimated using ordinary least squares (OLS), while the regressions presented in columns (3) and (4) were estimated using logit models*.

a *R^2^ for OLS regressions in columns (1) and (2) and pseudo R^2^ for logit regressions in columns (3) and (4)*.

In Column (2), we took risk preferences into account to ensure that risk preferences did not drive the effect of the levels of information on accepted prices. Both specifications show an insignificant effect of providing information about peers' decisions only and a significant, positive effect of providing information about both peers' decisions and feedback information about the optimal search duration on accepted prices in the search task. The coefficient on risk preferences was positive, as predicted by previous studies, though with only a moderate significance level (*p* = 0.062). None of the other control variables significantly impacted the accepted prices, except for the *Trial* dummies. As time passed, the subjects were more likely to search for higher prices. These results indicated that when a subject had access to both information about their peers' decisions and feedback information about the optimal search duration, he/she tended to accept a higher price, 9.35% higher than the subject who received no additional information in the single treatment, even when controlling for risk attitudes. This effect (73.512) is quite large because, on average, each subject changed their accepted price by 124.588 (either positively or negatively) per trial.

Since higher accepted prices do not always lead to more efficient searches, we defined the search behavior of a subject in one trial as an *Optimal search* if he/she stopped searching once a draw higher than or equal to 800 appeared, as described in the optimal search rule. We used the dummy variable, *Optimal search* for each subject in each trial as the dependent variable, and employed logit models^6^ to run the regressions. The results are presented in the last two columns of Table 3. These results are consistent with previous results and again confirm our findings. The significant coefficient of 0.523 indicates that holding all else equal, a subject who could observe feedback information about the optimal search duration and peers' real-time search decisions increased the odds ratio of conducting an optimal search by 68.7%,^7^ which showed a substantial treatment effect on the efficiency of the search from the pair-with-feedback treatment. The pair treatment, which provided the subjects with only information about their peers' decisions, did not improve the individuals' search efficiency.

**Result 3:** The presence of both real-time information about the decisions of peers and feedback information about the optimal search duration improved the accepted price and the efficiency of the search, but information about peers' decisions alone did not.

## Discussion and Conclusion

This study shows that peer effects exist in search behaviors when selling stocks under a pairwise grouping condition. People in a paired group exhibited significantly more similar search behaviors, particularly similar search durations, accepted prices, and incomes than people who are without peers. However, this peer effect disappeared in the presence of feedback information about the optimal search duration, perhaps because access to valuable information gave the subjects the confidence to search independently and ignore their peers' decisions. The subjects focused more on the differences between their own decisions and the optimal choice than comparing their choices with those of their peers. This finding indicates that peer effects work at least partially through the social learning channel. Recently, peer effects have been found in many decision-making situations (Cooper and Rege, 2011; Bursztyn et al., 2014; Beshears et al., 2015; Cai et al., 2015; Delfino et al., 2016; Lieber and Skimmyhorn, 2018). Further investigation into the moderation of peer effects using feedback techniques is necessary since both peers' decisions and feedback information are usually accessible and influence individuals' decisions simultaneously.

In addition, we explored whether learning from peers' decisions improved individuals' accepted prices and search efficiency. We found that information about peers' decisions alone did not increase individuals' accepted prices or optimize their search behaviors. However, such information did help when individuals were also able to observe feedback information about the optimal search duration, as was the condition in the pair-with-feedback treatment. In particular, observation of both feedback information about the optimal search duration and peers' real-time search decisions increased the accepted price by 9.35% and the odds ratio of conducting an optimal search by 68.7% compared to conditions in which no additional information was available, as was the condition in the single treatment. Peers' decisions alone had a negative but insignificant effect on the accepted prices and search efficiencies. This result again highlights the importance of feedback information for searching in the financial market, consistent with the findings of Einav (2005), who pointed out the positive effect of learning from post-purchase information on improving the efficiency of searches. Our result is inconsistent with the findings of some research on peer effects. For example, Falk and Ichino (2006) found that peer effects increased productivity by 16.3%. This inconsistency may reflect the different tasks used in the experiments. The task reported by Falk and Ichino (2006), stuffing letters into envelopes, was rather simple compared with our search task. Therefore, it appears that peer effects do not always have positive outcomes, especially in complex tasks such as making investment decisions.

Our results could have some implications for financial markets in the real world. Combining our results with those of previous related studies, we find that investors tend to imitate peers' investment decisions in both the process of buying and the process of selling risky assets. However, this imitation behavior is mitigated when investors can observe feedback information about the ex-post best responses. This is of great importance for financial policymakers attempting to decrease irrational herding and inefficient search behaviors. Policymakers should make more effort to facilitate the provision of more valuable information to investors. Such an improvement in the availability of information may nudge investors to make better investment choices, as suggested by nudge theory (Leonard et al., 2008).

Our study has some limitations. First, although the controlled experimental method ruled out most confounding factors, to a certain extent, it limited the external validity of our findings. There may be an experimenter demand effect in which subjects deliberately follow others because they believe that the experimenter expects to see herding. It is important to note that we used a neutral framework, by using points instead of prices, to illustrate our experiment to the subjects, which may have helped mitigate this potential demand effect. Employing a less direct way to present the decisions of peers can also be considered to further prevent this demand effect in the sense of making the objective of the experimenter less transparent to subjects. In addition, the experimental results still need to be developed before they can be extrapolated to the financial market. For example, we confirmed peer effects in stock price search behaviors under the pairwise grouping condition. One might be interested in whether our findings extend to larger social networks with an increased number of people. Second, the free acquisition of both pieces of information in our experiment is somewhat unrealistic. Information costs may influence individuals” imitations of peers during the selling process, which could be an interesting extension for future research.

In conclusion, we find that peer effects exist in stock price search behaviors but disappear when feedback information about the optimal search duration disappears. Observing both peers' decisions and feedback information about the optimal search duration improves the accepted price and the efficiency of searches, but information about peers' decisions alone does not.

## Data Availability Statement

The raw data supporting the conclusions of this article will be made available by the authors, without undue reservation.

## Ethics Statement

The studies involving human participants were reviewed and approved by Ethics committee for Key Laboratory of Applied Brain and Cognitive Sciences, Shanghai International Studies University. The participants provided their written informed consent to participate in this study.

## Author Contributions

XZ and YL performed the experiment and drew the figures. XZ, YL, and XY analyzed the data. All authors designed the experiment, wrote and revised the manuscript, and approved the version to be published.

## Conflict of Interest

The authors declare that the research was conducted in the absence of any commercial or financial relationships that could be construed as a potential conflict of interest.

## Footnotes

^1^Before the experiment, we used the computer to generate 50 realizations for 40 trials from the uniform distribution (0, 1000) and presented this realization to all of the subjects in the experiment. This method allowed us to control for sampling error and made the subjects' decisions comparable.

^2^We used the word “point” in favor of the neutral framework instead of “price” in the experimental instruction, hoping to mitigate the potential experimental demand effect.

^3^We used the exchange rate of 7.0647 CNY to 1 USD from October 27, 2019.

^4^Average search duration was calculated as the average number of rounds before a subject accepted the given point in 40 trials. The average accepted price was calculated as the average point accepted by the subject over 40 trials. The average income was calculated as the average payoff (the difference between accepted points and accumulated search costs were multiplied by the exchange rate, 0.015) over 40 trials.

^5^The number of possible configurations equals $\prod_{i=0}^{\left(N-2\right)/2}\left(N-2i-1\right)$ (Falk and Ichino, 2006), where *N* is the number of individuals, i.e., 20 in this case. Computing *Stdp* for all 654,729,075 configurations would require a large amount of computer time without substantially improving the reliability of our results. Therefore, we randomly chose 1/1000 of these configurations.

^6^Ordinary least squares estimations draw similar conclusions. We used logit models here to provide a more rigorous estimation when the dependent variable is binary.

^7^An estimated coefficient β in a logit model expresses the effect of the predictor variable on the log odds of the dependent variable. We can then calculate the effect on the odds ratio by inverting the logistic transformation, 100^*^(*e*^β^−1)%.
